# Proinflammatory Cytokines Trigger the Onset of Retinal Abnormalities and Metabolic Dysregulation in a Hyperglycemic Mouse Model

**DOI:** 10.1155/2023/7893104

**Published:** 2023-02-28

**Authors:** Gaganashree Shivashankar, Julie C. Lim, Monica L. Acosta

**Affiliations:** ^1^School of Optometry and Vision Science, New Zealand National Eye Centre and Centre for Brain Research, University of Auckland, Auckland, New Zealand; ^2^Department of Physiology, School of Medical and Health Sciences and New Zealand National Eye Centre, University of Auckland, Auckland, New Zealand

## Abstract

**Purpose:**

Recent evidence has shown that retinal inflammation is a key player in diabetic retinopathy (DR) pathogenesis. To further understand and validate the metabolic biomarkers of DR, we investigated the effect of intravitreal proinflammatory cytokines on the retinal structure, function, and metabolism in an in vivo hyperglycemic mouse model.

**Methods:**

C57Bl/6 mice were rendered hyperglycemic within one week of administration of a single high-dose intraperitoneal injection of streptozotocin, while control mice received vehicle injection. After confirming hyperglycemia, the mice received an intravitreal injection of either proinflammatory cytokines (TNF-*α* and IL-1*β*) or vehicle. Similarly, control mice received an intravitreal injection of either proinflammatory cytokines or vehicle. The retinal structure was evaluated using fundus imaging and optical coherence tomography, and retinal function was assessed using a focal electroretinogram (ERG), two days after cytokine injection. Retinas were collected for biochemical analysis to determine key metabolite levels and enzymatic activities.

**Results:**

Hyperglycemic mice intraocularly injected with cytokines developed visible retinal vascular damage and intravitreal and intraretinal hyper-reflective spots two days after the cytokines injection. These mice also developed a significant functional deficit with reduced a-wave and b-wave amplitudes of the ERG at high light intensities compared to control mice. Furthermore, metabolic disruption was evident in these mice, with significantly higher retinal glucose, lactate, ATP, and glutamine levels and a significant reduction in glutamate levels compared with control mice. Minimal or no metabolic changes were observed in hyperglycemic mice without intraocular cytokines or in control mice with intraocular cytokines at 2 days post hyperglycemia.

**Conclusions:**

Proinflammatory cytokines accelerated the development of vascular damage in the eyes of hyperglycemic mice. Significant changes were observed in retinal structure, function, and metabolic homeostasis. These findings support the idea that with the onset of inflammation in DR, there is a deficit in metabolism. Therefore, early intervention to prevent inflammation-induced retinal changes in diabetic patients may improve the disease outcome.

## 1. Introduction

Diabetic retinopathy (DR) is a progressive sight-threatening complication of diabetes that is clinically classified into early nonproliferative and late proliferative stages [1]. The current treatments halt the vascular damage in the late stage of DR. However, to prevent disease progression before the onset of significant visual dysfunction, novel treatment strategies should target the earlier molecular events that lead to vascular damage [2, 3].

Currently, the understanding is that not only hyperglycemia but also concomitant retinal inflammation is the major driving factors in the development of vascular abnormalities in DR [4–6]. A recent study from our group found that proinflammatory cytokines promote the development of severe vascular and functional abnormalities including vessel dilation, vessel beading, increased vessel tortuosity, and retinal oedema in the nonobese diabetic mice [7]. This demonstrated that the retina is susceptible to both hyperglycemia and proinflammatory conditions, affecting various interlinked metabolic pathways with a direct detrimental effect on retinal homeostasis [8]. In fact, previous studies have reported that the hyperglycemic retina undergoes apoptosis [9, 10], blood-retina barrier breakdown [11], activation of NF-*κ*B [10], and increased proliferation of microglial cells [12] only after inflammation becomes apparent.

Although current treatments such as panretinal photocoagulation, focal laser treatments, and intravitreal injection of anti-vascular endothelial growth factor (VEGF) target the late-stage sight-threatening vascular problems of DR, there is a high chance of reoccurrence of the disease [13–15]. Given that there has been significant advancement in an artificial intelligence-based system to detect clinical referable DR [16], our research contribution is the evaluation of early-stage disease to assess the consequences of retinal inflammation on retinal energy demand and on key metabolic pathways, which could be therapeutically modulated to delay the onset of vascular problems. Therefore, in this study, we investigated the morphological, functional, and biochemical outcomes of retinal inflammation in early streptozotocin (STZ)-induced diabetic mice with inflammation in the eye.

## 2. Materials and Methods

### 2.1. Animals

All animal experiments were approved by the University of Auckland Animal Ethics Committee (AEC 2205) and were conducted in accordance with the Association for Research in Vision and Ophthalmology (ARVO) statement on the use of animals in research. Six- to seven-week-old male C57BL/6 mice from the Vernon Jansen Unit, the vertebrate containment facility at the University of Auckland, were used in this study. Mice were bred and housed in standard cages under normal light-dark cycle conditions (12-h light (174 lux) and 12-h dark (<62 lux)) and had access to normal food and water *ad libitum*.

### 2.2. Development of the STZ-Induced Hyperglycemic Mouse Model

Baseline body weight and nonfasting blood glucose levels were measured before the induction of hyperglycemia. Body size and glucose measurements were obtained from eight mice that received a single 150 mg/kg body weight intraperitoneal injection of STZ prepared in 0.1 M sodium citrate as previously described [17]. Eight control mice received a sham intraperitoneal injection of 0.1 M sodium citrate. Nonfasting blood glucose levels were measured one week after administering STZ and repeated for three consecutive days to confirm hyperglycemia using a tail prick test. All blood glucose measurements were done consistently between 9.00 and 10.00 am. Mice with blood glucose levels higher than 15 mmol/L were considered hyperglycemic [18]. Blood glucose readings higher than the upper limit of detection (27.8 mmol/L) of the blood glucose meter (Freestyle Optium H Glucometer, UK) were recorded at 27.8 mmol/L for data analysis.

Although measurement of glycated hemoglobin level (HbA1c) is routinely used as an indicator to assess long-term glycaemic control, previous studies have reported that STZ-induced diabetic animals show a small, nonsignificant increase in HbA1C levels after 1 week of STZ injection [19, 20]. As the present study aims to investigate the early changes in the retina, it was unlikely that HbA1C levels would be significantly elevated after 1 week of STZ administration. Therefore, sustained hyperglycemic was confirmed by measuring blood glucose levels and HbA1C level was not evaluated in this study.

### 2.3. Intravitreal Injection of Proinflammatory Cytokines

An intravitreal injection of a proinflammatory cytokine cocktail was performed, as previously described [7]. The STZ or vehicle injection protocol was repeated to create control and hyperglycemic mice with or without intravitreal cytokines. Briefly, a group of hyperglycemic mice (*n* = 6) were intravitreally injected in both eyes with 500 ng/ml each of proinflammatory cytokine TNF-*α* (#RMTNFAI, Thermo Fisher Scientific, Waltham, MA), and 500 ng/ml IL-1*β* (#RMIL1BI; Thermo Fisher Scientific) in a total volume of 1 *μ*l (hyperglycemic mice with intraocular cytokines), while another group of six hyperglycemic mice received a vehicle (0.1 M PBS) intravitreal injection into both eyes (hyperglycemic group). Control mice received an intravitreal injection of proinflammatory cytokines (*n* = 6 control mice with intraocular cytokines) or a vehicle injection (*n* = 6 control mice, 12 eyes). Mugisho et al. (2018) observed that nonobese NOD diabetic mice develop clinical signs of DR as early as two days after intravitreal cytokine injection. Therefore, in this study, we investigated the structural, functional, and metabolic changes in the STZ-induced hyperglycemic mouse retina after two days of cytokine injection.

### 2.4. Fundus Imaging and Spectral Domain Optical Coherence Tomography (SD-OCT) Imaging

Fundus and SD-OCT imaging of the mouse retina was performed using the Micron IV imaging system as described previously [21, 22] to identify the appearance of clinical signs of DR including vessel beading and vessel tortuosity. Briefly, mice were anaesthetised by an intraperitoneal injection of ketamine (75 mg/kg body weight; Parnell Technologies, New Zealand) and domitor (0.5 mg/kg body weight; Zoetis, New Zealand). The pupils were dilated using 1% tropicamide (Bausch and Lomb New Zealand Ltd, New Zealand) and the cornea was maintained hydrated with 1% Poly Gel® lubricating eye gel (Alcon, Switzerland). The animals were then placed on a heating pad set at 37°C to maintain body temperature for the duration of anaesthesia. Eyes that developed cloudiness of the lens during anaesthesia were not possible to be analysed in the fundus and OCT studies. The fundus camera was carefully advanced towards the cornea until it came into contact with the poly gel. The Micron IV in-built StreamPix 6 software was used to visualise and record the retinal fundus. The retinal image was centered on the optic nerve head and blood vessel appearance was assessed. Vessel tortuosity was identified by the abnormal twists and turns of the blood vessels, and vessel beading was the alternating areas of blood vessel constriction that gave a “beaded appearance” to the blood vessels.

For the SD-OCT imaging, an ultrabroadband (160 nm) superluminescent diode centered at 830 nm was used as the light source to capture an image of 1024 pixels per A-scan with 1.8 *μ*m longitudinal resolutions. The horizontal line B-scan had 2 *μ*m axial resolutions. Each OCT scan consisted of twenty averaged B-scan images acquired at approximately one-disc diameter from the optic nerve head in the superior and inferior retinal quadrants. Images were analysed using ImageJ software (National Institute of Health, Maryland, USA) to evaluate retinal layer thickness.

### 2.5. Focal Electroretinography (ERG)

Retinal function was assessed using the image-guided focal ERG attachment of the Micron IV imaging system [23]. Briefly, mice were dark-adapted overnight and were handled under dim red-light illumination (*λ*_max_ = 650 nm). For the ERG recording, mice were anaesthetised by an intraperitoneal injection of ketamine (75 mg/kg body weight) and domitor (0.5 mg/kg body weight); the pupils were dilated using 1% tropicamide and the cornea was maintained hydrated with 1% Poly Gel® lubricating eye gel. The animal was positioned on the heating pad and a subdermal ground platinum electrode was inserted into the base of the tail and the reference platinum electrode was inserted under the scalp skin at the midpoint between the eyes. The objective lens containing the recording gold electrode was advanced towards the cornea and the retinal image under red-light illumination guided the area used to record the ERG.

A recording area of 0.75 mm in diameter was consistently chosen within the central superior retina, approximately one-disc diameter away from the optic nerve head. The ERG recordings were in response to white-light flashes from a light emitting diode (LED) source (5 millisecond duration) and were recorded using the LabScribeERG 3 software (Phoenix Research Labs). The light intensity used to elicit the ERG response ranged from −0.40 to 3.20 log candela seconds per square meter (log cd s/m^2^), with 20 sweeps and 10 second interval for −0.4 to 1.40 log cd s/m^2^, 10 sweeps and a 20 second interval for 2.00 log cd s/m^2^, 3 sweeps and a 60 second interval for 2.60 log cd s/m^2^, and 2 sweep and 2 second interval for 3.20 log cd s/m^2^. Multiple individual responses from each sweep were averaged to obtain an improved signal-to-noise ratio [24]. One eye (left or right) per animal was used in the study. The ERG responses were recorded from the superior retina as this was the quadrant mostly associated with retinal thinning during DR [25–28]. The a-wave, b-wave, and implicit time as well as summed OP response were measured using the Micron IV software.

### 2.6. Biochemical Assays

For these biochemical assays data was collected from one or both eyes per experimental group. Retinal metabolites were measured using commercially available kits: glucose (Glucose-Glo™ Assay, #J6021, Promega, Madison, USA), lactate (Lactate-Glo™ Assay, #J5021, Promega), glutamate-glutamine (Glutamine/Glutamate-Glo™ Assay, #J8021, Promega) and adapted to analysis of the retina according to the manufacturer's protocol. Briefly, the retinal glutamate and glutamine levels were measured by incubating the sample with and without glutaminase for 40 minutes, followed by a 60-minute incubation with the supplier's glutamate detection reagent in 1 : 1 ratio. The luminescence signal generated by the assay was recorded using the EnSpire® Multimode Plate Reader (Perkin Elmer, Massachusetts, USA). A glutamate standard curve was used to determine the concentration of glutamate and glutamine in the retinal supernatant. The retinal glucose and lactate levels were determined similarly, wherein the sample was incubated with the respective detection reagent and the luminescence signal was recorded. Glucose, lactate, glutamate, and glutamine levels are reported as nmol/mg protein.

ATP levels were measured using the Adenosine 5′-triphosphate (ATP) Bioluminescent Assay Kit (#FLAA, Sigma–Aldrich, Missouri, USA) according to the manufacturer's protocol. Briefly, a freshly prepared ATP assay mix was added to the retinal supernatant and the luminescence signal was recorded immediately. The ATP levels are reported as pmol/mg protein.

### 2.7. Enzymatic Activities in the Retina

Enzymatic activity was evaluated using retinal samples from six eyes per animal group. Glutamine synthetase (GS) activity was evaluated using the method employed in a previous study [29]. Briefly, the reaction mixture was prepared with 50 mM Imidazole-HCl buffer (pH 7.1), 7.6 mM ATP, 1.0 mM phosphoenolpyruvate, 50 mM MgCl_2_, 10 mM KCl, 40 mM NH_4_Cl_2_, 0.35 mM NADH, 0.1 M monosodium glutamate, 25 *μ*g of pyruvate kinase, and 50 *μ*g of lactate dehydrogenase in a volume of 1.0 ml. To eliminate traces of ADP and pyruvate, the reaction mixture was equilibrated at 30°C for 10 minutes. The retinal samples were added to the reaction mixture in a 1 : 1 ratio and the rate of change in NADH absorbance was measured at 340 nm, 37°C using the EnSpire® Multimode Plate Reader for 10 minutes. The specific enzyme activity of GS was normalised to total retinal protein concentration and expressed as µmoles per minute per milligram protein.

The glyceraldehyde 3-phosphate dehydrogenase (GAPDH) activity Assay kit (#ab204732, Abcam, Australia) was used to evaluate GAPDH enzyme activity. In this assay, GAPDH catalyses the conversion of glyceraldehyde-3-phosphate to 1,3-bisphosphate glycerate resulting in a stoichiometric NADH generation, which further reacts with the developer to form a coloured product with an absorbance maximum at 450 nm. The retina was homogenised in GAPDH assay buffer and centrifuged at 10,000 × g for 5 minutes at 4°C to remove any cellular debris. The retinal supernatant was added to the reaction mix containing GAPDH assay buffer, GAPDH developer, and GAPDH substrate in a 1 : 1 ratio and the colorimetric change was measured kinetically every 5 minutes, for a total of 20 minutes at 450 nm using a plate reader. A NADH standard curve was generated to calculate the specific GAPDH activity. GAPDH activity was expressed as nmoles per minute per milligram of protein.

### 2.8. Statistical Analysis

Normality of biochemical data distribution was confirmed using the Shapiro–Wilk normality test and QQ plots (see appendix). The statistical significance for focal ERGs was determined using two-way ANOVA and a post hoc Dunnett's multiple comparison tests. Statistical significance for the biochemical assays was determined using one-way ANOVA and a post hoc Dunnett's multiple comparison tests. The added effect of cytokines over high glucose conditions alone was evaluated by an unpaired *t*-test. A *p* value of less than 0.05 was considered statistically significant. The statistical analysis was conducted using six-eight eyes per animal group. The “*n*” value in the figure legend indicates individual retinas, with at least six per experimental group. All statistical analysis was performed using GraphPad Prism 8.

As both eyes from the same animal were used for some experiments, sensitivity analysis using the generalised estimating equations was performed to confirm the outcome obtained through traditional statistics. This analysis was performed using IBM SPSS statistics software (version 29).

## 3. Results

### 3.1. STZ-Induced Hyperglycemic Mice had Reduced Body Weight and Elevated Blood Glucose Levels within One Week

**Figure 1:**
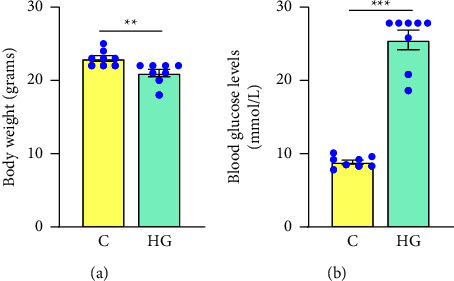
Development of the hyperglycemic mouse model. Body weight and blood glucose levels were measured before and after the STZ injection. STZ administration caused a significant decrease in body weight (a) and a significant increase in blood glucose levels (b). The unpaired *t*-test test was used to determine statistical significance. Values represent mean ± SEM (*n* = 8 mice per group). ^∗∗^*p* < 0.01, ^∗∗∗^*p* < 0.001. Abbreviation- C: control mice, HG: hyperglycemic mice.

Body weight and nonfasting blood glucose levels were determined before and after STZ injection (Figure 1). One week after STZ administration, there was a significant 9% (*p* = 0.006) decrease in body weight in all STZ-injected animals and nonfasting blood glucose levels were significantly elevated by an average of 188% (*p* = 0.0002), ranging from 18.6 to 27.8 mmol/L in hyperglycemic mice compared to control mice (7.8 to 10.1 mmol/L).

### 3.2. Proinflammatory Cytokines-Induced Vascular Changes in the Hyperglycemic Mouse Retina

**Figure 2:**
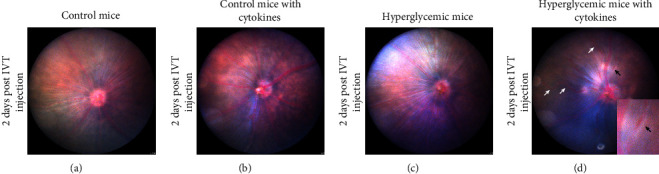
Representative fundus images showing changes in retinal vasculature two days after intravitreal injection. Fundus image of control mice (a), control mice with intraocular cytokines (b), hyperglycemic mice without intraocular cytokines (c), and hyperglycemic mice with intraocular cytokines (d). Intravitreal injection of proinflammatory cytokines caused blood vessel beading (indicated by black arrow, inset) and increased vessel tortuosity (indicated by white arrows) in hyperglycemic mice.

Fundus examination was performed two days after the intravitreal injection, and the appearance of the retinal vasculature was assessed (Figure 2). Control mice (Figure 2(a)), control mice with intraocular cytokines (Figure 2(b)) as well as hyperglycemic mice without intraocular cytokines (Figure 2(c)) did not show signs of blood vessel damage or changes in the retinal vasculature. However, intravitreal injection of cytokines to hyperglycemic mice resulted in blood vessel tortuosity in three out of ten eyes and blood vessel beading in five out of ten eyes two days after the intravitreal injection (Figure 2(d)).

### 3.3. Proinflammatory Cytokines-Induced Retinal and Vitreous Hyper-Reflective Spots in Hyperglycemic Mice

**Figure 3:**
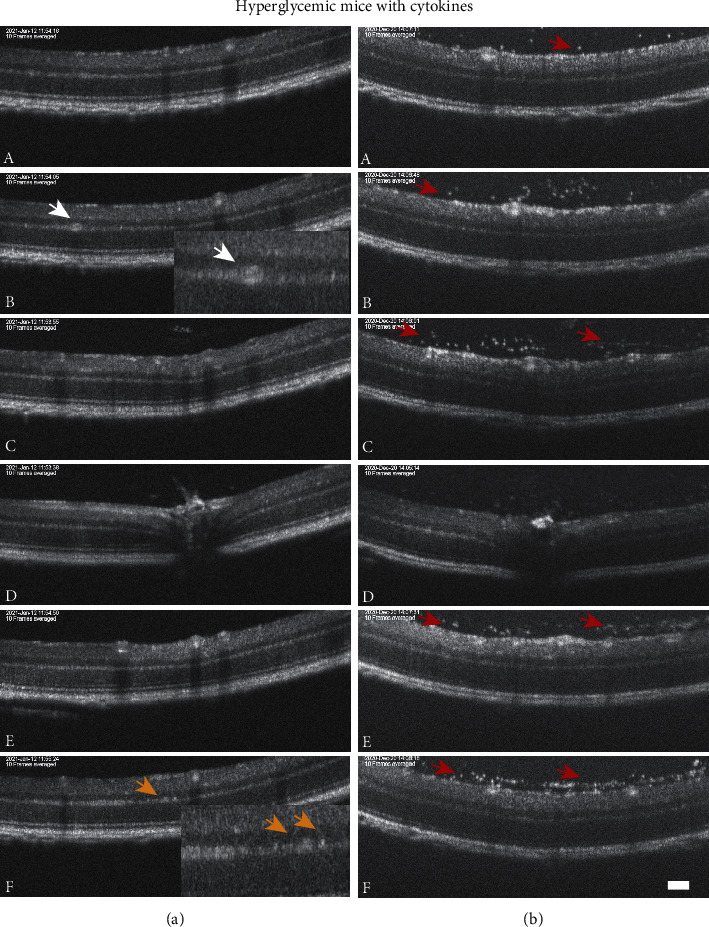
Representative SD-OCT images of the retina two days after intravitreal cytokine injection. The serial OCT images (A-F) in two hyperglycemic mice injected with cytokines are shown in (a) and (b). Hyperglycemic mice with intraocular cytokines developed retinal hyper-reflective spots (spots less than 20 *μ*m indicated by orange arrows and spots greater than 50 *μ*m indicated by the white arrows) primarily within the inner nuclear layer and outer plexiform layer. The hyperglycemic mice with intraocular cytokines also developed vitreal hyper-reflective spots (indicated by red arrows). The A-F images were acquired at the position of the green scan lines in the fundus image shown in Supplementary Figure 3. Scale bar is 100 *μ*m.

SD-OCT imaging was performed to evaluate structural changes in the retina two days after intravitreal injection (Figure 3). The OCT scans showed that both hyperglycemic mice with and without intraocular cytokines developed distinct hyper-reflective spots 2 days after the intravitreal injection. Two out of eight eyes (25%) of the hyperglycemic mice without intraocular cytokines and seven out of ten eyes (70%) of the hyperglycemic mice with intraocular cytokines developed small (less than 20 *μ*m) hyper-reflective spots, while six out of ten eyes (60%) of the hyperglycemic mice with intraocular cytokines developed large (greater than 50 *μ*m) hyper-reflective spots (Figure 3). These hyper-reflective spots were located near the outer plexiform layer and within the inner nuclear layer. Furthermore, three out of ten eyes (30%) of the hyperglycemic mice with intraocular cytokines developed vitreal hyper-reflective spots (Figure 3(g)), which was not observed in any of the hyperglycemic mice without intraocular cytokines. Intravitreal cytokine injection did not induce any retinal abnormalities in control mice. Retinal layer thickness measurements confirmed no retinal thinning in any of the groups compared to control mice (Supplementary Figure 2).

### 3.4. Proinflammatory Cytokines Caused Retinal Functional Deficit in Hyperglycemic Mice

**Figure 4:**
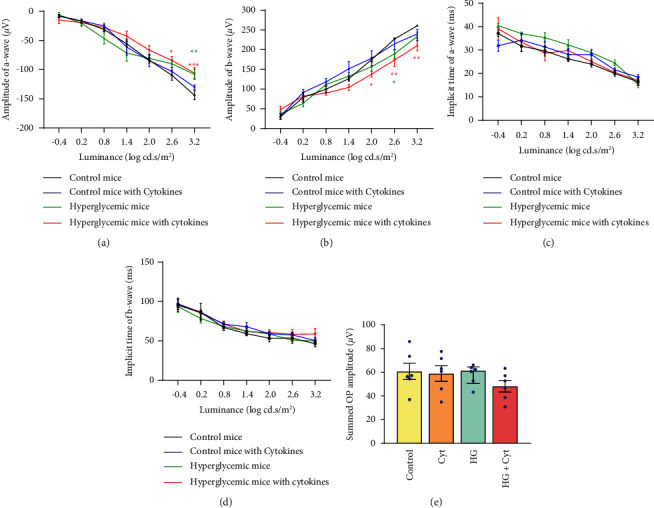
Focal ERG a-wave and b-wave amplitude and implicit time two days after intravitreal injection. (a) Average a-wave amplitude. (b) Average b-wave amplitude. (c) Average a-wave implicit time. (d) Average b-wave implicit time. (e) Summed OP amplitude. Two days postintravitreal injection, there was a significant reduction in a-wave amplitude in the hyperglycemic mice at 3.2 log cd.s/m^2^ (represented by the green asterisk) compared to control, while the hyperglycemic mice with intraocular cytokines showed further reduction of the a-wave amplitude at 2.6 and 3.2 log cd.s/m^2^ (represented by the red asterisk) compared to control. Likewise, there was a significant reduction in b-wave amplitude in the hyperglycemic mice at 2.6 log cd.s/m^2^ (represented by the green asterisk) compared to control, while the hyperglycemic mice with intraocular cytokines showed further reduced b-wave amplitude at 2.0, 2.6, and 3.2 log cd.s/m^2^ (represented by the red asterisk) compared to control. There were no significant changes in the OP amplitude in the experimental group compared to control mice. Statistical significance was determined using two-way ANOVA followed by post hoc Dunnett's multiple comparison tests. Values represent mean ± SEM (*n* = 6). ^∗^*p* < 0.05; ^∗∗^*p* < 0.01; ^∗∗∗^*p* < 0.001.

**Figure 5:**
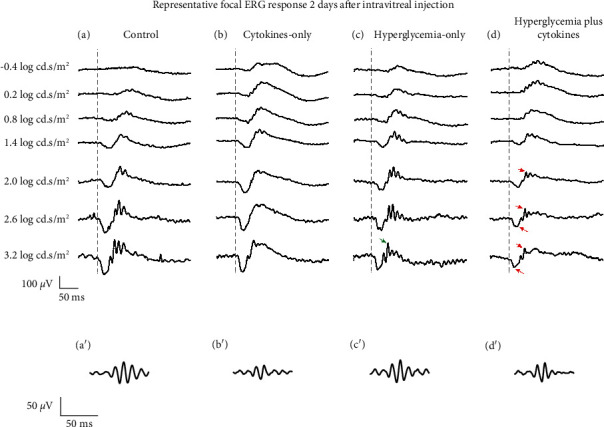
Representative focal ERG response two days after intravitreal injection. Focal ERG response in control mice (a), control mice intravitreally injected with cytokines (b), hyperglycemic mice without intraocular cytokines (c), and hyperglycemic mice with intraocular cytokines (d). a′, b′, c′, and d′ are the OPs within the corresponding ERG response at the highest light intensity (3.2 log cd.s/m2). The a-wave, b-wave, and the OP response of the control mice to intraocular cytokines were comparable to the control mice. While the b-wave amplitude of the hyperglycemic mice without intravitreal cytokines appeared to be reduced (indicated in green arrow), the hyperglycemic mice with intraocular cytokines had visibly reduced a-wave and b-wave amplitudes at 2, 2.6, and 3.2 log cd.s/m2 (indicated in red arrows). The control and hyperglycemic mice injected with proinflammatory cytokines appeared to have low OP response (b′ and d′) compared to control mice. The vertical grey dashed line at 50 ms represents the time point at which the white-light stimulus is presented.

Focal ERG response was recorded to evaluate retinal function two days after intravitreal injection. Hyperglycemic mice with and without cytokines had a significant reduction in the ERG a-wave and b-wave amplitudes compared to control mice. The a-wave amplitude of hyperglycemic mice without cytokines was not reduced significantly except at light intensity 3.2 log cd s/m^2^ (two-way ANOVA, *p* = 0.033, Figure 4(a)) and the b-wave amplitude was not reduced except at light intensity 2.6 log cd s/m^2^ (*p* = 0.030, Figures 5(c) and 4(b)). Whereas, intravitreal injection of proinflammatory cytokines to hyperglycemic mice significantly further reduced the a-wave amplitude at light intensities 2.6 (*p* = 0.001) and 3.2 log cd s/m^2^ (*p* = 0.0007, Figures 5(d) and 4(a)) and reduced the b-wave amplitude at light intensities 2.0 log cd s/m^2^ (*p* = 0.032), 2.6 log cd s/m^2^ (*p* = 0.001) and 3.2 log cd s/m^2^ (*p* = 0.002, Figures 5(d) and 4(b)) compared to control mice. No significant changes were observed in the a-wave (Figure 4(c)) and b-wave (Figure 4(d)) implicit times in all conditions.

There were no statistically significant differences in the summed OP response in any experimental mouse group compared to control mice (Figure 4(e)), although the individual OP response appeared to be reduced in hyperglycemic mice injected with proinflammatory cytokines.

### 3.5. Proinflammatory Cytokines Trigger a Progressive Loss of Metabolic Homeostasis in Hyperglycemic Mice

**Figure 6:**
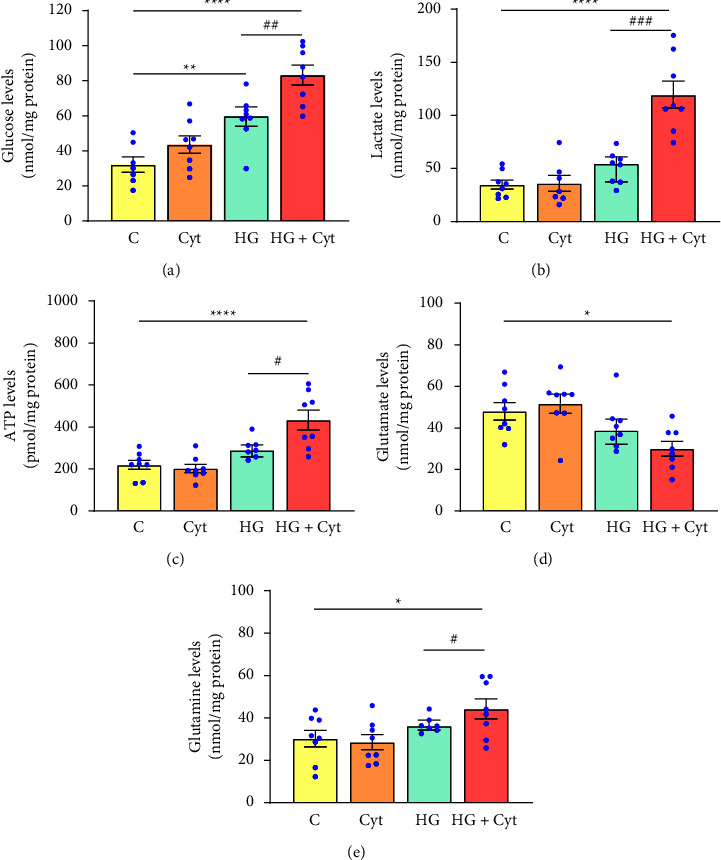
Metabolite levels in the retina two days after intravitreal injection. (a) Glucose levels, (b) lactate levels, (c) ATP levels, (d) glutamate levels, and (e) glutamine levels. Although hyperglycemic mice without cytokines showed significantly high retinal glucose levels, intravitreal injection of cytokines to hyperglycemic mice further increased glucose, lactate, ATP, glutamine levels, and reduced glutamate levels compared to control mice (represented by the asterisk). Moreover, intravitreal injection of cytokines to hyperglycemic mice caused a significant change in glucose, lactate, ATP, and glutamine levels compared to hyperglycemic mice without intraocular cytokines (represented by the hashtag). One-way ANOVA followed by post hoc Dunnett's multiple comparison tests was used to determine statistical significance compared to controls. The unpaired *t*-test was used to determine the added effect of cytokines on hyperglycemic mice. Values represent mean ± SEM (*n* = 6–8 eyes). ^∗^ and ^#^*p* < 0.05, ^∗∗^ and ^##^*p* < 0.01, ^∗∗∗^ and ^###^*p* < 0.001, ^∗∗∗∗^*p* < 0.0001. Abbreviations- C: control mice, Cyt: mice with cytokines, HG: hyperglycemic mice, HG + Cyt: hyperglycemic mice with cytokines.

The metabolic status of the DR retina was assessed by evaluating key metabolite levels two days postintravitreal injection. Retinal glucose levels were elevated by 81% (*p* = 0.003) in hyperglycemic mice without cytokines compared with control mice, and those with intraocular cytokines had a significantly elevated retinal glucose of 158% (*p* < 0.0001), lactate levels by 243% (*p* < 0.0001), ATP by 97% (*p* < 0.0001), glutamine levels by 54% (*p* = 0.004), and reduced glutamate levels by 37% (*p* = 0.011) compared to control mice (Figure 6). Intravitreal cytokine injection to hyperglycemic mice further increased glucose levels by 42% (*p* = 0.005), lactate levels by 134% (*p* = 0.0002), ATP levels by 45% (*p* = 0.025), glutamine levels by 27% (*p* = 0.029) above the levels observed in hyperglycemic mice without cytokines.

**Figure 7:**
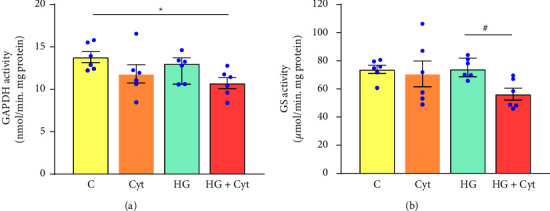
Enzymatic activity in the retina two days after intravitreal injection. (a) Glyceraldehyde 3-phosphate dehydrogenase (GAPDH) activity. (b) Glutamine synthetase (GS) activity. Hyperglycemic mice with intraocular cytokines showed significantly reduced GAPDH activity compared to control mice. Although the GS activity in hyperglycemic mice with intraocular cytokines remained unchanged compared to control mice, intravitreal injection of cytokines to hyperglycemic mice caused a significant decrease in GS activity compared to hyperglycemic mice without intraocular cytokines. One-way ANOVA followed by post hoc Dunnett's multiple comparison tests was used to determine statistical significance. The unpaired *t*-test was used to determine the added effect of cytokines on hyperglycemic mice. Values represent mean ± SEM (*n* = 6 eyes). ^*∗*^ and # represent *p* < 0.05. Abbreviation- C: control mice, Cyt: mice with cytokines, HG: hyperglycemic mice, HG + Cyt: hyperglycemic mice with cytokines.

Hyperglycemic mice with intraocular cytokines showed significantly reduced GAPDH activity by 20% (*p* = 0.031), while GS activity remained unchanged compared to control mice. However, intravitreal injection of cytokines to hyperglycemic mice caused a significant decrease in GS activity by 24% (*p* = 0.004) compared to hyperglycemic mice without cytokines (Figure 7).

## 4. Discussion

In this study, it was confirmed that proinflammatory cytokines trigger the development of vascular and retinal abnormalities and metabolic dysregulation characteristic of DR in a STZ-induced hyperglycemic mouse model. The STZ-induced diabetic mouse model is one of the most widely used diabetic models [30], and significant DR-related retinal and vascular signs were evident once exposed to a proinflammatory environment. In the STZ mouse model, proinflammatory cytokines were delivered as a single injection, and combined with hyperglycemia, this single dose of proinflammatory cytokines leads to ocular vascular damage within two days. Hyperglycemic mice did not develop any severe ocular vascular abnormalities for the duration of this study and confirmed that STZ-induced diabetic mice rarely develop serious vascular abnormalities until 6 to 12 month posthyperglycemia [31–33]. The effects of STZ-induced hyperglycemia are seen after 6 months, when retinal physiological and biochemical changes are observed [33]. However, the intravitreal administration of proinflammatory cytokines to hyperglycemic mice accelerated the onset of changes, and resulted in vessel beading, increased vessel tortuosity, and retinal and vitreal hyper-reflective spots within two days. These vascular changes are considered early indicators of microvascular damage in DR [34] and were in line with previous findings from Mugisho et al. (2018) [7], wherein intravitreal cytokine injection to nonobese diabetic (NOD) mice triggered the development of severe vascular abnormalities within one week. The development of retinal and vitreal hyper-reflective spots is in concurrence with previous findings in patients with early DR [35, 36] and in the nonobese NOD DR mouse model [7] and DR rat model [37]. These previous studies have shown that intraretinal hyper-reflective spots in the inner retina could be associated with microaneurysms and macroaneurysms based on their size [37, 38], while other studies have demonstrated that the appearance of hyper-reflective spots in the inner nuclear layer is a definitive marker of vascular abnormality [35, 38]. Although we did not collect histological sections for the hyper-reflective spots in this model, hyper-reflective spots were found to be associated with activated microglial cells that are responsible for mediating the early inflammatory response in DR [35, 39–41]. Moreover, the precise location of these hyper-reflective spots in the retina can be predictive of disease progression, as microglial cells tend to migrate from the inner retina towards the photoreceptors with time in the diabetic retina [35]. Although less frequent, vitreal hyper-reflective spots are indicative of a more severe form of DR and are commonly reported in patients with severe proliferative DR [42]. These vitreal hyper-reflective spots are considered infiltrating macrophages that contribute to endothelial cell damage and eventually cause vascular breakdown in DR [43–45]. Hence, low-grade, subclinical inflammation seems to be a critical factor for the development of vascular lesions in DR [7, 43].

In addition to vascular and retinal abnormalities, intravitreal injection of cytokines triggered retinal functional deficits in hyperglycemic mice. Our findings were consistent with previous reports [46, 47] that reduced ERG a-wave and b-wave amplitudes indicative of impairment of photoreceptor and inner retinal function were early events in DR pathology. Although photoreceptors are generally not considered affected in DR (most likely due to the substantial distance from the retinal vasculature), recent reports have found that they are important mediators of oxidative stress and inflammation in DR [48, 49]. In fact, it was observed that patients with retinitis pigmentosa, a rare genetic disorder causing photoreceptor degeneration, were less likely to develop DR associated retinal complications in diabetic patients [50, 51]. Moreover, in addition to Müller cells and microglia, photoreceptors were found to be major contributors of oxidative stress and inflammation in the retina during DR [48] and were found to be apoptotic in STZ-induced diabetic rats [52]. Hence, early metabolic dysregulation in the photoreceptors and within the downstream inner retinal neurons [53] are the likely cause of the reduced a-wave and b-wave amplitudes in DR. However, in spite of the reduced a-wave and b-wave responses, the summed OPs remained unaffected in these mice, implying that the inhibitory feedback pathway initiated in the inner retina by the amacrine cells remained unaffected [54]. However, we hypothesize that significant change in OP amplitude becomes apparent with increasing DR duration.

Altered retinal function is often indicative of an underlying metabolic imbalance [55, 56]. Our previous studies have confirmed that co-exposure of mouse retinal explants to hyperglycemia and proinflammatory cytokines causes biochemical and neurochemical changes in retinal neurons and Müller cells, wherein the metabolite levels of glucose, lactate, ATP, and glutamate are altered, in addition to the re-distribution of glutamate and glutamine within the inner retinal neurons [57, 58]. As expected, hyperglycemic mice with intraocular cytokines showed altered glucose, lactate, ATP, glutamate, and glutamine levels, suggesting that cytokines can trigger metabolic dysregulation in hyperglycemic mice within two days. In addition, we also found that the activities of GAPDH and GS were altered in hyperglycemic mice with intraocular cytokines, and these enzymes are critical in regulating glucose and glutamate-regulating pathways [58]. This implies that inflammation could be the causative factor of early functional deficits in DR. We observed the same outcome in an in vitro model of high glucose plus cytokines [58], where lactate accumulation may also be mediated by pyruvate recycling when GAPDH activity is reduced [59, 60] or shunting through AGE pathways [61]. There is evidence of complete oxidative degradation of glutamate to form pyruvate and then lactate via a nonglycolytic route [62]. In support of this, previous studies have reported that metabolic pathways including glycolysis [63–65] and glutamate pathways [56], alter retinal function. These early functional deficits were also reported in diabetic patients with no apparent DR signs and without any significant visual dysfunction, suggesting that functional deficits may precede the manifestation of clinical signs of DR but are more likely to be a strong indicator of DR severity and the magnitude of functional loss [66]. Although retinal thinning is a clinical marker of neurodegeneration and is an early occurrence in DR [28, 67], no changes in the retinal layer thickness at both time points were observed, suggesting that functional and metabolic changes in DR may precede the occurrence of neurodegeneration.

Low glutamate and high glutamine levels observed in our model were not due to elevated glutamine synthetase activity. In such a scenario, glutamate hypersensitivity (a cause for retinal damage) is possible as overall decrease in glutamate reflects a rapid decrease in its synthesis from carbon dioxide, rather than from glutamine as described by Gowda et al. [68] in diabetic conditions. Our previous studies showed this decrease in glutamate in bipolar cells [58] and confirmed the decrease in glutamate and increase in glutamine. We have also shown that despite low glutamate levels, there are specific retinal areas of glutamate accumulation in Müller endfeet, and this would be the areas of glutamate hypersensitivity in DR. Thus, we think that glutamine levels reflect the state of the diabetic retina, but we do not have evidence that glutamine accumulation is damaging the retina.

Rajagopal et al. (2016) have shown that development and progression of DR in fat-fed mice are also associated with hyperglycemia and inflammation. Functional ocular deficits were characterised by electroretinographic dysfunction observed at beginning of 6 months due to glucose intolerance with microvascular disease appearing at 12 months. Interestingly, inflammasome activation was reported at 3 months, before the development of systemic glucose intolerance, electroretinographic defects, or microvascular disease. These results reinforce our suggestion that disease in the diabetic environment may progress through inflammatory stages long before the development of vascular lesions [69].

Therefore, the findings from our study emphasise the importance of early intervention targeting mediators of inflammation to slow down or prevent the progression of DR. In support of this, recent studies have shown that metabolic inhibitors such as the polymethoxylated flavone Niboletin [70] and a Chinese herbal formula, Shuangdan Mingmu capsule [71], promote the upregulation of GAPDH activity in DR, while resveratrol treatment [72] prevents GS downregulation in DR. These metabolic inhibitors could potentially prevent blood-retina barrier breakdown, oxidative stress-induced apoptosis of pericytes, and glutamate excitotoxicity in DR. Moreover, immunological therapy is fast gaining popularity to treat retinal inflammation in DR [73, 74], and drugs of particular interest are the Connexin-43 hemichannel inhibitors. Previous study from our group has shown that Connexin-43 hemichannel blocker mitigates retinal inflammation by effectively blocking the NLRP3 inflammasome pathway in an in vivo mouse model of DR [37, 75, 76].

In conclusion, this study is consistent with the hypothesis that proinflammatory cytokines aggravate the early morphological, functional, and metabolic imbalance in hyperglycemic mice and opens up the opportunity for a wider array of possible therapies concomitantly targeting inflammation and early metabolic dysregulation in DR. Moreover, these findings suggest that early visual dysfunction precedes retinal neurodegeneration and the appearance of severe vascular pathology in DR and are indicative of alerted retinal bioenergetics. Therefore, we have compiled evidence to suggest that it is crucial to control retinal inflammation in diabetics to prevent or delay the rapid worsening of retinal metabolism, which may trigger functional changes and may progress with time if not treated early on.

## Data Availability

The data supporting the findings of this study are included in the figure and supplementary data.

## Supplementary Materials

Supplementary Materials
*Supplementary Figure 1*. Normality tests, *p* > 0.05 on the Shapiro–Wilk test is indicative of a distribution of data that is not statistically significant from a normal distribution. *Supplementary Figure 2*. Retinal layer thickness measured two days after intravitreal injection. The ratio of retinal layer thickness to the total retinal thickness was consistently measured in the central retina. (A) NFL-GCL-IPL layer thickness ratio. (B) INL layer thickness ratio. (C) OPL layer thickness ratio. (D) ONL layer thickness ratio. (E) IS/OS layer thickness ratio. (F) RPE-Choroid layer thickness ratio. No change in the retinal layer thickness was observed in experimental mice compared to control mice 2 days after intravitreal injection. One-way ANOVA followed by post hoc Dunnett's multiple comparison tests was used to determine significance. Values represent mean ± SEM (*n* = 6 eyes). Abbreviation- (C) control mice, Cyt: mice with intraocular cytokines, HG: hyperglycemic mice, HG + Cyt: hyperglycemic mice with intraocular cytokines, NFL-GCL-IPL: nerve fiber layer-ganglion cell layer-inner plexiform layer, INL: inner nuclear layer, OPL: outer plexiform layer, ONL: outer nuclear layer, IS/OS: inner-outer segments, RPE: retinal pigment epithelium. *Supplementary Figure 3*. Representative SD-OCT images of the control, control mice with cytokines and hyperglycemic mice retina. (A) The thin green OCT scan line in the fundus image of the retina represents the exact location at which the cross-sectional OCT images (A–F) were taken. (B) Representative OCT image showing the retinal layers. Abbreviation- NFL-GCL-IPL: nerve fiber layer-ganglion cell layer-inner plexiform layer, INL: inner nuclear layer, OPL: outer plexiform layer, ONL: outer nuclear layer, IS/OS: inner-outer segments, RPE: retinal pigment epithelium.

## References

[1] Bandello F., Zarbin M. A., Lattanzio R. (2014). *Clinical Strategies in the Management of Diabetic Retinopathy*.

[2] Whitehead M., Wickremasinghe S., Osborne A., Van Wijngaarden P., Martin K. R. (2018). Diabetic retinopathy: a complex pathophysiology requiring novel therapeutic strategies. *Expert Opinion on Biological Therapy*.

[3] Valdez-Guerrero A. S., Quintana-Pérez J. C., Arellano-Mendoza M. G., Castañeda-Ibarra F. J., Tamay-Cach F., Alemán-González-Duhart D. (2020). Diabetic retinopathy: important biochemical alterations and the main treatment strategies. *Canadian Journal of Diabetes*.

[4] Kastelan S., Tomic M., Gverović Antunica A., Salopek Rabatić J., Ljubic S. (2013). Inflammation and pharmacological treatment in diabetic retinopathy. *Mediators of Inflammation*.

[5] McGuire P., Rangasamy S., Das A. (2012). Diabetic retinopathy and inflammation: novel therapeutic targets. *Middle East African Journal of Ophthalmology*.

[6] Tang J., Kern T. S. (2011). Inflammation in diabetic retinopathy. *Progress in Retinal and Eye Research*.

[7] Mugisho O. O., Rupenthal I. D., Squirrell D. M. (2018). Intravitreal pro-inflammatory cytokines in non-obese diabetic mice: modelling signs of diabetic retinopathy. *PLoS One*.

[8] Roy S., Kern T. S., Song B., Stuebe C. (2017). Mechanistic insights into pathological changes in the diabetic retina: implications for targeting diabetic retinopathy. *American Journal Of Pathology*.

[9] Joussen A. M., Doehmen S., Le M. L. (2009). TNF-alpha mediated apoptosis plays an important role in the development of early diabetic retinopathy and long-term histopathological alterations. *Molecular Vision*.

[10] Kowluru R. A., Odenbach S. (2004). Role of interleukin-1*β* in the pathogenesis of diabetic retinopathy. *British Journal of Ophthalmology*.

[11] Huang H., Gandhi J. K., Zhong X. (2011). TNF*α* is required for late BRB breakdown in diabetic retinopathy, and its inhibition prevents leukostasis and protects vessels and neurons from apoptosis. *Investigative Opthalmology and Visual Science*.

[12] Baptista F. I., Aveleira C. A., Castilho ÁF., Ambrósio A. F. (2017). Elevated glucose and interleukin-1*β* differentially affect retinal microglial cell proliferation. *Mediators of Inflammation*.

[13] Abu El-Asrar A. M., Al-Mezaine H. S. (2011). Advances in the treatment of diabetic retinopathy. *Saudi journal of ophthalmology*.

[14] Chappelow A. V., Tan K., Waheed N. K., Kaiser P. K. (2012). Panretinal photocoagulation for proliferative diabetic retinopathy: pattern scan laser versus argon laser. *American Journal of Ophthalmology*.

[15] Osaadon P., Fagan X. J., Lifshitz T., Levy J. (2014). A review of anti-VEGF agents for proliferative diabetic retinopathy. *Eye*.

[16] Xie L., Yang S., Squirrell D., Vaghefi E. (2020). Towards implementation of AI in New Zealand national diabetic screening program: cloud-based, robust, and bespoke. *PLoS One*.

[17] Zhang Y., Peng T., Zhu H. (2010). Prevention of hyperglycemia-induced myocardial apoptosis by gene silencing of toll-likereceptor-4. *Journal of Translational Medicine*.

[18] Kohzaki K., Vingrys A. J., Bui B. V. (2008). Early inner retinal dysfunction in streptozotocin-induced diabetic rats. *Investigative Opthalmology and Visual Science*.

[19] Fujii E., Nomoto T. (1984). Changes in glycosylated hemoglobin in short-and semi long-termstreptozotocin-diabetic mice and rats. *The Japanese Journal of Pharmacology*.

[20] Nagisa Y., Kato K., Watanabe K. (2003). Changes in glycated haemoglobin levels in diabetic rats measured with an automatic affinity HPLC. *Clinical and Experimental Pharmacology and Physiology*.

[21] Guo C. X., Mat Nor M. N., Danesh-Meyer H. V. (2016). Connexin43 mimetic peptide improves retinal function and reduces inflammation in a light-damaged albino rat model. *Investigative Opthalmology and Visual Science*.

[22] Mat Nor N., Guo C. X., Rupenthal I. D., Chen Y. S., Green C. R., Acosta M. L. (2018). Sustained Connexin43 mimetic peptide release from loaded nanoparticles reduces retinal and choroidal photodamage. *Investigative Opthalmology and Visual Science*.

[23] Becker S., Wang H., Simmons A. B. (2018). Targeted knockdown of overexpressed VEGFA or VEGF164 in muller cells maintains retinal function by triggering different signaling mechanisms. *Scientific Reports*.

[24] Holopigian K., Hood D. C. (2003). Electrophysiology. *Opthalmology Clinics of North America*.

[25] Gundogan F. C., Akay F., Uzun S., Yolcu U., Çağıltay E., Toyran S. (2015). Early neurodegeneration of the inner retinal layers in type 1 diabetes mellitus. *Ophthalmologica*.

[26] Luan H., Roberts R., Sniegowski M., Goebel D. J., Berkowitz B. A. (2006). Retinal thickness and subnormal retinal oxygenation response in experimental diabetic retinopathy. *Investigative Opthalmology and Visual Science*.

[27] Sugimoto M., Sasoh M., Ido M., Wakitani Y., Takahashi C., Uji Y. (2005). Detection of early diabetic change with optical coherence tomography in type 2 diabetes mellitus patients without retinopathy. *Ophthalmologica*.

[28] Toprak I., Fenkci S. M., Fidan Yaylali G., Martin C., Yaylali V. (2019). Early retinal neurodegeneration in preclinical diabetic retinopathy: a multifactorial investigation. *Eye*.

[29] Kingdon H. S., Hubbard J. S., Stadtman E. R. (1968). Regulation of glutamine synthetase. XI. the nature and implications of a lag phase in the escherichia coli glutamine synthetase reaction. *Biochemistry*.

[30] Lai A. K. W., Lo A. C. Y. (2013). Animal models of diabetic retinopathy: summary and comparison. *Journal of Diabetes Research*.

[31] Feit-Leichman R. A., Kinouchi R., Takeda M. (2005). Vascular damage in a mouse model of diabetic retinopathy: relation to neuronal and glial changes. *Investigative Opthalmology and Visual Science*.

[32] Li Q., Verma A., Han P. Y. (2010). Diabetic eNOS-knockout mice develop accelerated retinopathy. *Investigative Opthalmology and Visual Science*.

[33] Robinson R., Barathi V. A., Chaurasia S. S., Wong T. Y., Kern T. S. (2012). Update on animal models of diabetic retinopathy: from molecular approaches to mice and higher mammals. *Disease models and mechanisms*.

[34] Sasongko M. B., Wong T. Y., Nguyen T. T., Cheung C. Y., Shaw J. E., Wang J. J. (2011). Retinal vascular tortuosity in persons with diabetes and diabetic retinopathy. *Diabetologia*.

[35] Vujosevic S., Bini S., Midena G., Berton M., Pilotto E., Midena E. (2013). Hyperreflective intraretinal spots in diabetics without and with nonproliferative diabetic retinopathy: an in vivo study using spectral domain OCT. *Journal of Diabetes Research*.

[36] Arthi M., Sindal M., Rashmita R. (2021). Hyperreflective foci as biomarkers for inflammation in diabetic macular edema: retrospective analysis of treatment naïve eyes from south India. *Indian Journal of Ophthalmology*.

[37] Mat Nor M. N., Rupenthal I. D., Green C. R., Acosta M. L. (2020). Connexin hemichannel block using orally delivered tonabersat improves outcomes in animal models of retinal disease. *Neurotherapeutics*.

[38] Soriano M. E. T., Aguirre G. G. (2016). *Diagnostic Atlas of Retinal Diseases*.

[39] Vujosevic S., Midena E. (2013). Retinal layers changes in human preclinical and early clinical diabetic retinopathy support early retinal neuronal and müller cells alterations. *Journal of Diabetes Research*.

[40] Vujosevic S., Bini S., Torresin T. (2017). Hyperreflective retinal spots in normal and diabetic eyes: B-scan and en face spectral domain optical coherence tomography evaluation. *Retina*.

[41] Vujosevic S., Toma C. (2018). Diabetic retinopathy: an inflammatory disease. *Annals of Eye Science*.

[42] Mizukami T., Hotta Y., Katai N. (2017). Higher numbers of hyperreflective foci seen in the vitreous on spectral-domain optical coherence tomographic images in eyes with more severe diabetic retinopathy. *Ophthalmologica*.

[43] Joussen A. M., Murata T., Tsujikawa A., Kirchhof B., Bursell S. E., Adamis A. P. (2001). Leukocyte-mediated endothelial cell injury and death in the diabetic retina. *American Journal Of Pathology*.

[44] Saito M., Barbazetto I. A., Spaide R. F. (2013). Intravitreal cellular infiltrate imaged as punctate spots by spectral-domain optical coherence tomography in eyes with posterior segment inflammatory disease. *Retina*.

[45] Kokona D., Häner N. U., Ebneter A., Zinkernagel M. S. (2017). Imaging of macrophage dynamics with optical coherence tomography in anterior ischemic optic neuropathy. *Experimental Eye Research*.

[46] Sergeys J., Etienne I., Van Hove I. (2019). Longitudinal in vivo characterization of the streptozotocin-induced diabetic mouse model: focus on early inner retinal responses. *Investigative Opthalmology and Visual Science*.

[47] Becker S., Carroll L. S., Vinberg F. (2020). Rod phototransduction and light signal transmission during type 2 diabetes. *BMJ open diabetes research and care*.

[48] Du Y., Veenstra A., Palczewski K., Kern T. S. (2013). Photoreceptor cells are major contributors to diabetes-induced oxidative stress and local inflammation in the retina. *Proceedings of the National Academy of Sciences*.

[49] Kern T. S., Berkowitz B. A. (2015). Photoreceptors in diabetic retinopathy. *Journal of diabetes investigation*.

[50] Arden G. B. (2001). The absence of diabetic retinopathy in patients with retinitis pigmentosa: implications for pathophysiology and possible treatment. *British Journal of Ophthalmology*.

[51] de Gooyer T. E., Stevenson K. A., Humphries P., Simpson D. A. C., Gardiner T. A., Stitt A. W. (2006). Retinopathy is reduced during experimental diabetes in a mouse model of outer retinal degeneration. *Investigative Opthalmology and Visual Science*.

[52] Park S. H., Park J. W., Park S. J. (2003). Apoptotic death of photoreceptors in the streptozotocin-induced diabetic rat retina. *Diabetologia*.

[53] Yumnamcha T., Guerra M., Singh L. P., Ibrahim A. S. (2020). Metabolic dysregulation and neurovascular dysfunction in diabetic retinopathy. *Antioxidants*.

[54] Wachtmeister L. (1998). Oscillatory potentials in the retina: what do they reveal. *Progress in Retinal and Eye Research*.

[55] Bui B. V., Kalloniatis M., Vingrys A. J. (2004). Retinal function loss after monocarboxylate transport inhibition. *Investigative Opthalmology and Visual Science*.

[56] Bui B. V., Hu R. G., Acosta M. L., Donaldson P., Vingrys A. J., Kalloniatis M. (2009). Glutamate metabolic pathways and retinal function. *Journal of Neurochemistry*.

[57] Shivashankar G., Lim J. C., Acosta M. L. (2020). Proinflammatory cytokines trigger biochemical and neurochemical changes in mouse retinal explants exposed to hyperglycemic conditions. *Molecular Vision*.

[58] Shivashankar G., Lim J. C., Acosta M. L. (2021). Glyceraldehyde-3-phosphate dehydrogenase and glutamine synthetase inhibition in the presence of pro-inflammatory cytokines contribute to the metabolic imbalance of diabetic retinopathy. *Experimental Eye Research*.

[59] Dienel G. A., McKenna M. C. (2014). A dogma‐breaking concept: glutamate oxidation in astrocytes is the source of lactate during aerobic glycolysis in resting subjects. *Journal of Neurochemistry*.

[60] Luo X., Li R., Yan L. J. (2015). Roles of pyruvate, NADH, and mitochondrial complex I in redox balance and imbalance in *β* cell function and dysfunction. *Journal of Diabetes Research*.

[61] de Arriba S. G., Loske C., Meiners I. (2003). Advanced glycation endproducts induce changes in glucose consumption, lactate production, and ATP levels in SH-SY5Y neuroblastoma cells by a redox-sensitive mechanism. *Journal of Cerebral Blood Flow and Metabolism*.

[62] Sonnewald U. (2014). Glutamate synthesis has to be matched by its degradation - where do all the carbons go?. *Journal of Neurochemistry*.

[63] Noell W. K. (1951). The effect of iodoacetate on the vertebrate retina. *Journal of Cellular and Comparative Physiology*.

[64] Winkler B. S. (1981). Glycolytic and oxidative metabolism in relation to retinal function. *The Journal of General Physiology*.

[65] Ames A., Li Y. Y., Heher E. C., Kimble C. R. (1992). Energy metabolism of rabbit retina as related to function: high cost of na+ transport. *Journal of Neuroscience*.

[66] Miller D. J., Cascio M. A., Rosca M. G. (2020). Diabetic retinopathy: the role of mitochondria in the neural retina and microvascular disease. *Antioxidants*.

[67] Sohn E. H., van Dijk H. W., Jiao C. (2016). Retinal neurodegeneration may precede microvascular changes characteristic of diabetic retinopathy in diabetes mellitus. *Proceedings of the National Academy of Sciences of the United States of America*.

[68] Gowda K., Zinnanti W. J., LaNoue K. F. (2011). The influence of diabetes on glutamate metabolism in retinas. *Journal of Neurochemistry*.

[69] Rajagopal R., Bligard G. W., Zhang S., Yin L., Lukasiewicz P., Semenkovich C. F. (2016). Functional deficits precede structural lesions in mice with high-fatdiet-induced diabetic retinopathy. *Diabetes*.

[70] Miyata Y., Matsumoto K., Kusano S. (2021). Regulation of endothelium-reticulum-stress-mediated apoptotic cell death by a polymethoxylated flavone, nobiletin, through the inhibition of nuclear translocation of glyceraldehyde 3-phosphate dehydrogenase in retinal müller cells. *Cells*.

[71] Nie F., Yan J., Ling Y. (2021). Effect of shuangdan mingmu capsule, a Chinese herbal formula, on oxidative stress-induced apoptosis of pericytes through PARP/GAPDH pathway. *BMC complementary medicine and therapies*.

[72] Zeng K., Yang N., Wang D. (2016). Resveratrol prevents retinal dysfunction by regulating glutamate transporters, glutamine synthetase expression and activity in diabetic retina. *Neurochemical Research*.

[73] Al Mamun A., Mimi A. A., Zaeem M. (2021). Role of pyroptosis in diabetic retinopathy and its therapeutic implications. *European Journal of Pharmacology*.

[74] Takeda A., Yanai R., Murakami Y., Arima M., Sonoda K. H. (2020). New insights into immunological therapy for retinal disorders. *Frontiers in Immunology*.

[75] Mugisho O. O., Rupenthal I. D., Paquet-Durand F., Acosta M. L., Green C. R. (2019). Targeting connexin hemichannels to control the inflammasome: the correlation between connexin43 and NLRP3 expression in chronic eye disease. *Expert Opinion on Therapeutic Targets*.

[76] Louie H. H., Shome A., Kuo C. Y., Rupenthal I. D., Green C. R., Mugisho O. O. (2021). Connexin43 hemichannel block inhibits NLRP3 inflammasome activation in a human retinal explant model of diabetic retinopathy. *Experimental Eye Research*.

