# Supplementary figures and images for: Proinflammatory Cytokines Trigger the Onset of Retinal Abnormalities and Metabolic Dysregulation in a Hyperglycemic Mouse Model
Source: J Ophthalmol. 2023 Feb 28;2023:7893104. doi: 10.1155/2023/7893104 (PMC9991478; doi:10.1155/2023/7893104)

**
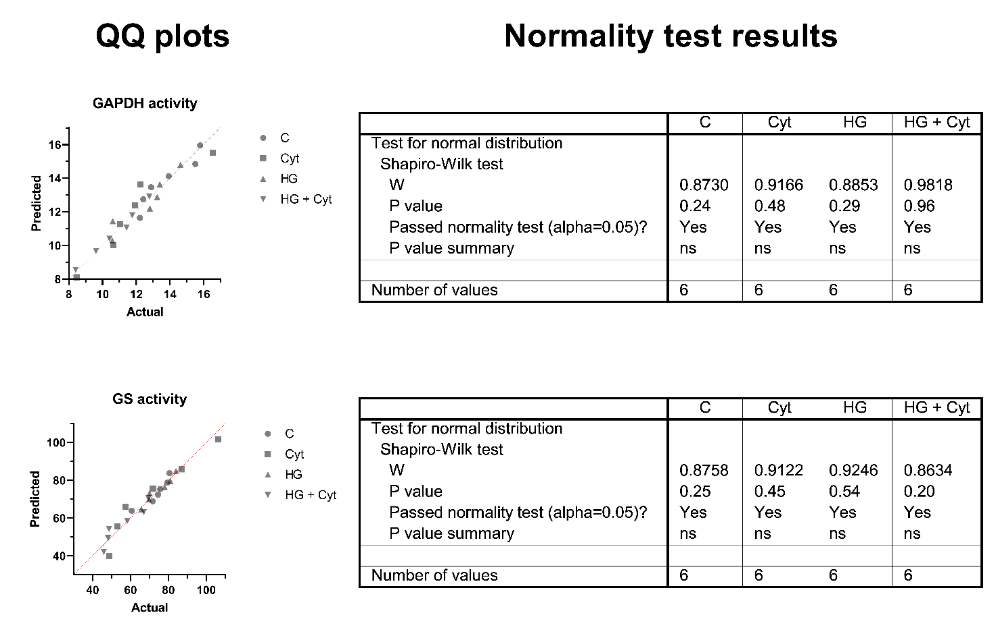
**
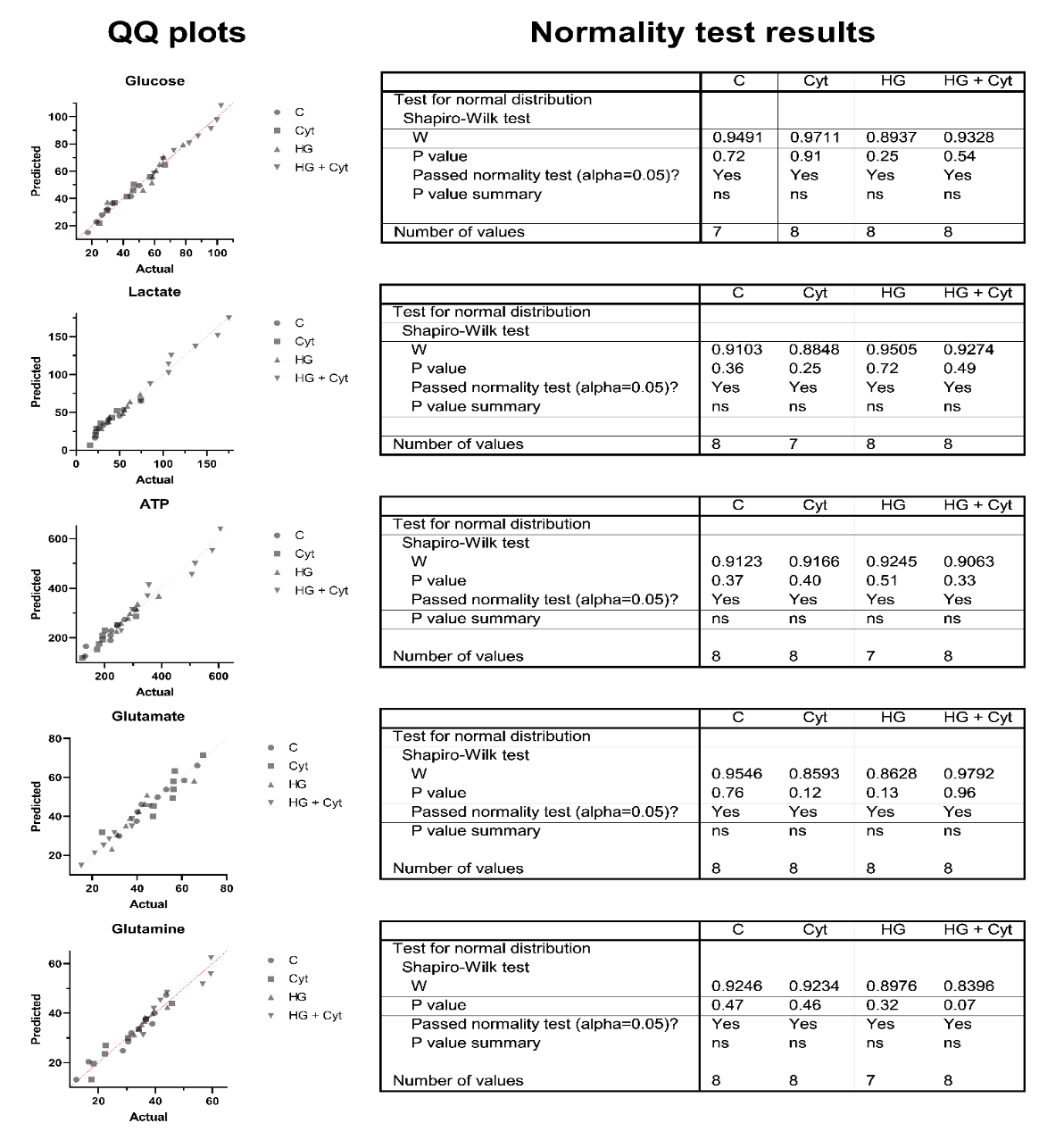

Supplement: Supplementary Materials — Supplementary Figure 1. Normality tests, p > 0.05 on the Shapiro–Wilk test is indicative of a distribution of data that is not statistically significant from a normal distribution. Supplementary Figure 2. Retinal layer thickness measured two days after intravitreal injection. The ratio of retinal layer thickness to the total retinal thickness was consistently measured in the central retina. (A) NFL-GCL-IPL layer thickness ratio. (B) INL layer thickness ratio. (C) OPL layer thickness ratio. (D) ONL layer thickness ratio. (E) IS/OS layer thickness ratio. (F) RPE-Choroid layer thickness ratio. No change in the retinal layer thickness was observed in experimental mice compared to control mice 2 days after intravitreal injection. One-way ANOVA followed by post hoc Dunnett's multiple comparison tests was used to determine significance. Values represent mean ± SEM (n = 6 eyes). Abbreviation- (C) control mice, Cyt: mice with intraocular cytokines, HG: hyperglycemic mice, HG + Cyt: hyperglycemic mice with intraocular cytokines, NFL-GCL-IPL: nerve fiber layer-ganglion cell layer-inner plexiform layer, INL: inner nuclear layer, OPL: outer plexiform layer, ONL: outer nuclear layer, IS/OS: inner-outer segments, RPE: retinal pigment epithelium. Supplementary Figure 3. Representative SD-OCT images of the control, control mice with cytokines and hyperglycemic mice retina. (A) The thin green OCT scan line in the fundus image of the retina represents the exact location at which the cross-sectional OCT images (A–F) were taken. (B) Representative OCT image showing the retinal layers. Abbreviation- NFL-GCL-IPL: nerve fiber layer-ganglion cell layer-inner plexiform layer, INL: inner nuclear layer, OPL: outer plexiform layer, ONL: outer nuclear layer, IS/OS: inner-outer segments, RPE: retinal pigment epithelium. [file 7893104.f1.zip › Supplementary figure 1 (1).docx]

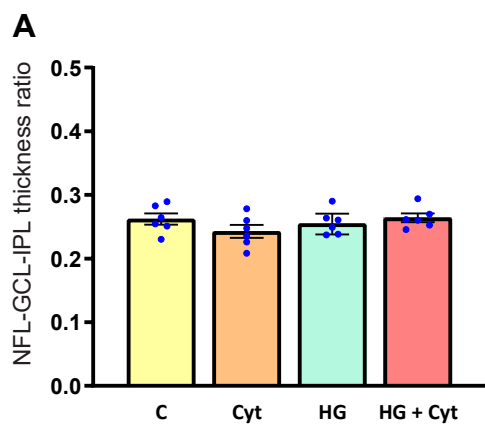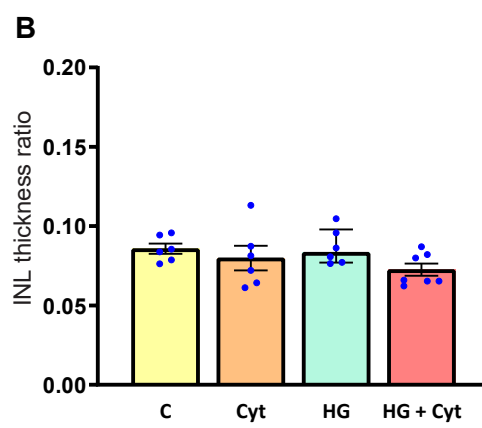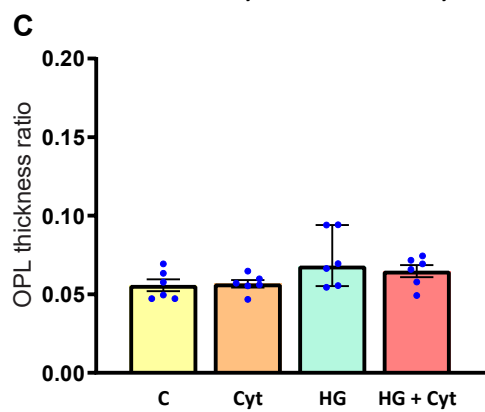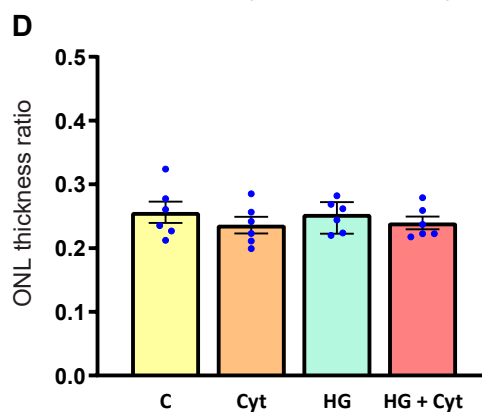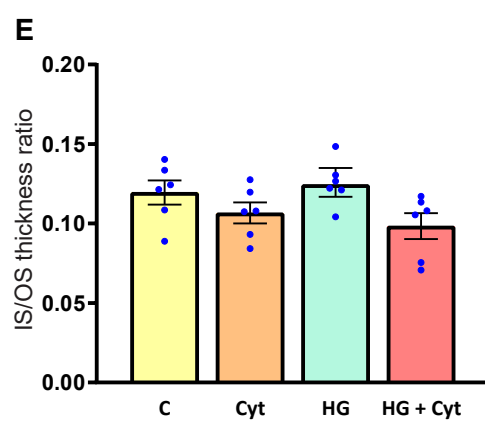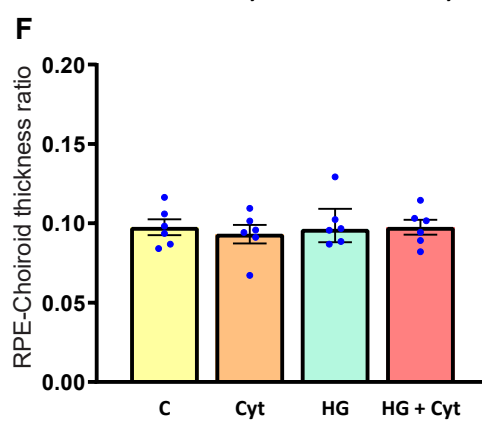

Supplement: Supplementary Materials — Supplementary Figure 1. Normality tests, p > 0.05 on the Shapiro–Wilk test is indicative of a distribution of data that is not statistically significant from a normal distribution. Supplementary Figure 2. Retinal layer thickness measured two days after intravitreal injection. The ratio of retinal layer thickness to the total retinal thickness was consistently measured in the central retina. (A) NFL-GCL-IPL layer thickness ratio. (B) INL layer thickness ratio. (C) OPL layer thickness ratio. (D) ONL layer thickness ratio. (E) IS/OS layer thickness ratio. (F) RPE-Choroid layer thickness ratio. No change in the retinal layer thickness was observed in experimental mice compared to control mice 2 days after intravitreal injection. One-way ANOVA followed by post hoc Dunnett's multiple comparison tests was used to determine significance. Values represent mean ± SEM (n = 6 eyes). Abbreviation- (C) control mice, Cyt: mice with intraocular cytokines, HG: hyperglycemic mice, HG + Cyt: hyperglycemic mice with intraocular cytokines, NFL-GCL-IPL: nerve fiber layer-ganglion cell layer-inner plexiform layer, INL: inner nuclear layer, OPL: outer plexiform layer, ONL: outer nuclear layer, IS/OS: inner-outer segments, RPE: retinal pigment epithelium. Supplementary Figure 3. Representative SD-OCT images of the control, control mice with cytokines and hyperglycemic mice retina. (A) The thin green OCT scan line in the fundus image of the retina represents the exact location at which the cross-sectional OCT images (A–F) were taken. (B) Representative OCT image showing the retinal layers. Abbreviation- NFL-GCL-IPL: nerve fiber layer-ganglion cell layer-inner plexiform layer, INL: inner nuclear layer, OPL: outer plexiform layer, ONL: outer nuclear layer, IS/OS: inner-outer segments, RPE: retinal pigment epithelium. [file 7893104.f1.zip › Supplementary figure 2.pdf]

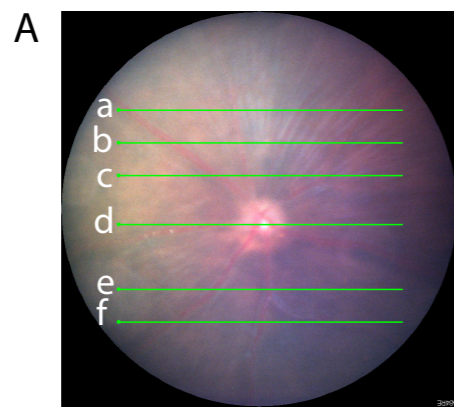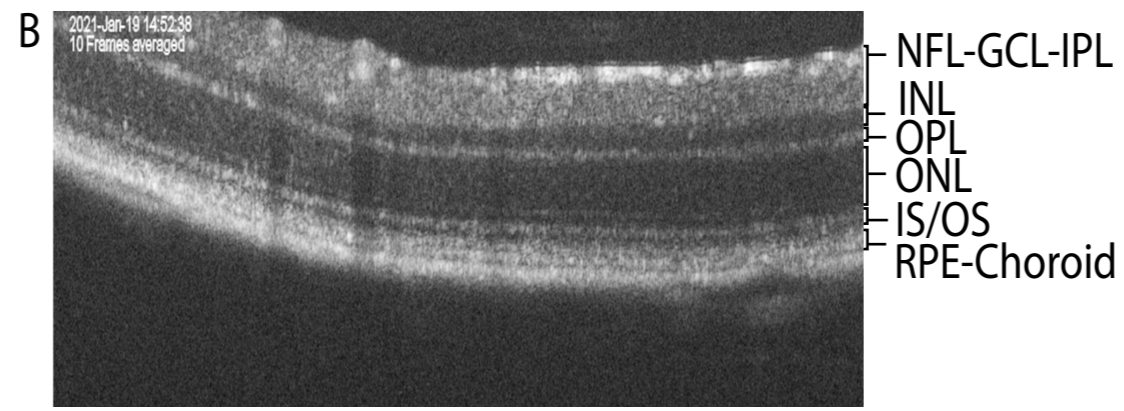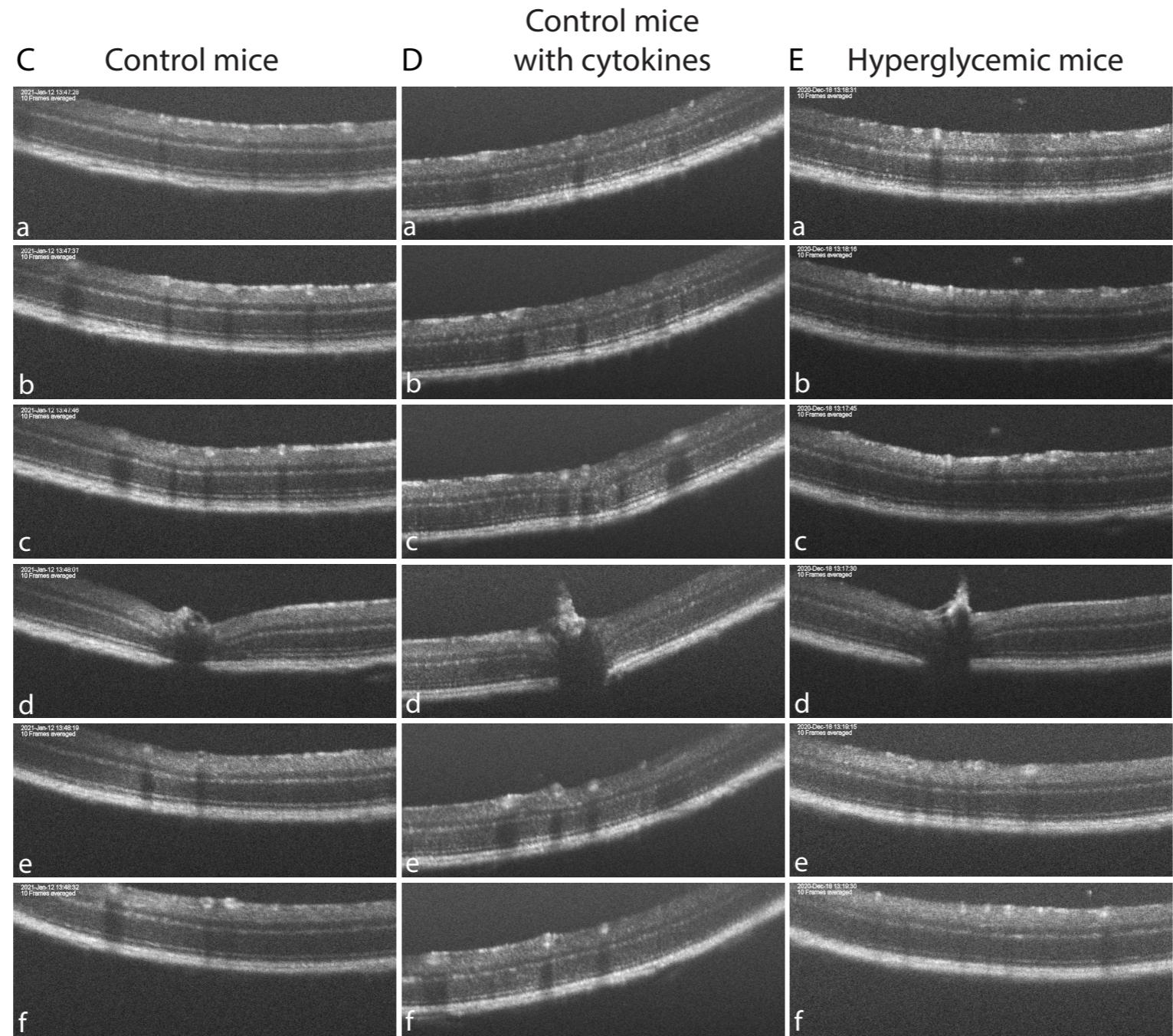

Supplement: Supplementary Materials — Supplementary Figure 1. Normality tests, p > 0.05 on the Shapiro–Wilk test is indicative of a distribution of data that is not statistically significant from a normal distribution. Supplementary Figure 2. Retinal layer thickness measured two days after intravitreal injection. The ratio of retinal layer thickness to the total retinal thickness was consistently measured in the central retina. (A) NFL-GCL-IPL layer thickness ratio. (B) INL layer thickness ratio. (C) OPL layer thickness ratio. (D) ONL layer thickness ratio. (E) IS/OS layer thickness ratio. (F) RPE-Choroid layer thickness ratio. No change in the retinal layer thickness was observed in experimental mice compared to control mice 2 days after intravitreal injection. One-way ANOVA followed by post hoc Dunnett's multiple comparison tests was used to determine significance. Values represent mean ± SEM (n = 6 eyes). Abbreviation- (C) control mice, Cyt: mice with intraocular cytokines, HG: hyperglycemic mice, HG + Cyt: hyperglycemic mice with intraocular cytokines, NFL-GCL-IPL: nerve fiber layer-ganglion cell layer-inner plexiform layer, INL: inner nuclear layer, OPL: outer plexiform layer, ONL: outer nuclear layer, IS/OS: inner-outer segments, RPE: retinal pigment epithelium. Supplementary Figure 3. Representative SD-OCT images of the control, control mice with cytokines and hyperglycemic mice retina. (A) The thin green OCT scan line in the fundus image of the retina represents the exact location at which the cross-sectional OCT images (A–F) were taken. (B) Representative OCT image showing the retinal layers. Abbreviation- NFL-GCL-IPL: nerve fiber layer-ganglion cell layer-inner plexiform layer, INL: inner nuclear layer, OPL: outer plexiform layer, ONL: outer nuclear layer, IS/OS: inner-outer segments, RPE: retinal pigment epithelium. [file 7893104.f1.zip › Supplementary figure 3.pdf]
